# Cyto-Feature Engineering: A Pipeline for Flow Cytometry Analysis to Uncover Immune Populations and Associations with Disease

**DOI:** 10.1038/s41598-020-64516-0

**Published:** 2020-05-06

**Authors:** Amy Fox, Taru S. Dutt, Burton Karger, Mauricio Rojas, Andrés Obregón-Henao, G. Brooke Anderson, Marcela Henao-Tamayo

**Affiliations:** 1 Mycobacteria Research Laboratories, Department of Microbiology, Immunology, and Pathology, Colorado State University, Fort Collins, Colorado 80523 USA; 2 Grupo de Inmunología Celular e Inmunogenética, Facultad de Medicina, Unidad de Citometría de Flujo, Sede de Investigación Universitaria, Universidad de Antioquia, Medellin, Colombia; 3 Department of Environmental and Radiological Health Sciences, Colorado State University, Fort Collins, Colorado 80523 USA

**Keywords:** Infectious diseases, Vaccines, Immunology, Microbiology

## Abstract

Flow cytometers can now analyze up to 50 parameters per cell and millions of cells per sample; however, conventional methods to analyze data are subjective and time-consuming. To address these issues, we have developed a novel flow cytometry analysis pipeline to identify a plethora of cell populations efficiently. Coupled with feature engineering and immunological context, researchers can immediately extrapolate novel discoveries through easy-to-understand plots. The R-based pipeline uses Fluorescence Minus One (FMO) controls or distinct population differences to develop thresholds for positive/negative marker expression. The continuous data is transformed into binary data, capturing a positive/negative biological dichotomy often of interest in characterizing cells. Next, a filtering step refines the data from all identified cell phenotypes to populations of interest. The data can be partitioned by immune lineages and statistically correlated to other experimental measurements. The pipeline’s modularity allows customization of statistical testing, adoption of alternative initial gating steps, and incorporation of other datasets. Validation of this pipeline through manual gating of two datasets (murine splenocytes and human whole blood) confirmed its accuracy in identifying even rare subsets. Lastly, this pipeline can be applied in all disciplines utilizing flow cytometry regardless of cytometer or panel design. The code is available at https://github.com/aef1004/cyto-feature_engineering.

## Introduction

Flow cytometers can now analyze up to 50 parameters (antigens, size, granularity, cytokines, transcription factors, etc.) per cell and millions of cells per sample^1^. Conventional flow cytometry data analysis uses manual gating of cells on 2D plots to distinguish populations 1–2 dimensions at a time; this makes it both subjective and time consuming (up to 15 hours per experiment)^2^. Better methods are therefore critically needed to take full advantage of this powerful technology. Researchers have responded with open-source tools, including tools for automated gating to remove user input bias (e.g., *openCyto*) and tools to identify and cluster cell populations concurrently using all parameters (e.g., *FlowSOM*, t-SNE)^3–5^. While powerful advances, these new tools lack a straightforward way to integrate data from important technical controls or to compare resulting cell populations with other experimental measurements. Work is ongoing across several research groups to extend existing open-source tools to address some of these gaps. *CytoCompare* and *cytofast*, for example, focus on data analysis after clustering^6,7^. However, few tools exist that allow users to incorporate the many flow cytometry controls required for good data acquisition and analysis, and the output from the available clustering tools are often difficult for immunologists to interpret.

We have developed an end-to-end method for analyzing flow cytometry data that aims to address these limitations. For flow cytometry data, a parameter often represents a biologically binary phenomenon—that a marker is present or missing on a cell. While variation exists in the flow cytometry measurements for each parameter within cells in each binary group, that within-group variation is often uninformative noise. Our pipeline leverages this underlying biology—it uses feature engineering to create binary features for whether each cell has a positive or negative value for each marker. It does this using either external thresholds identified based on Fluorescence Minus One controls (FMOs) or the availability to separate the data based on clear population separation. The pipeline therefore identifies cell populations based on positive/negative combinations of each flow cytometry marker, a description that is readily interpretable by immunologists.

In four main steps, the pipeline: (1) cleans the data for live, single cells; (2) feature engineers the data based on FMO cutoffs or population separation; (3) analyzes the flow cytometry samples for all populations present in the sample and filters to populations above a population size threshold; (4) visualizes resulting populations through heatmaps of cell phenotypes and time series plots within experimental groups. Furthermore, it allows the use of statistical testing to identify cell populations associated with other experimental measurements (e.g., disease burden as measured through colony forming units) and novel populations induced by any experimental or clinical condition. All steps in the pipeline are modular, allowing each to be modified or replaced depending on the research question and features of the experimental data. As a case study, we illustrate the pipeline on a study involving *Mycobacterium bovis* Bacillus Calmette-Guérin (BCG)-vaccinated or control (Phosphate buffered saline (PBS)-injected) C57BL/6 mice infected with *Mycobacterium tuberculosis (M. tuberculosis)*. We further validate the pipeline analyzing human whole blood for B and T cells.

## Results

**Figure 1:**
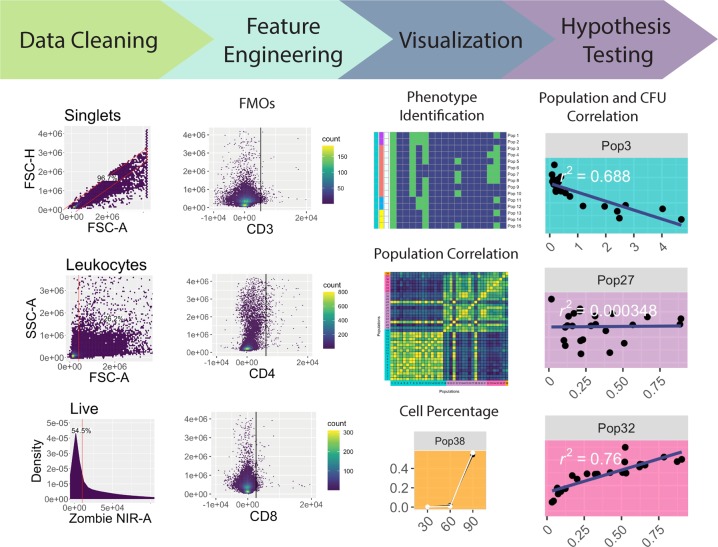
Pipeline workflow. Initial data cleaning is performed on all FMOs and samples. A singlet gate on the root population gates out doublets (top panel under “Data Cleaning”). Debris is removed from the samples with a “mindensity” gate from the R package *openCyto* (middle panel under “Data Cleaning”), and then the live cells (those negative for Zombie NIR) are gated, also using a “mindensity” gate (bottom panel under “Data Cleaning”). The data is then feature engineered into binary data based on FMOs. It is then filtered to a smaller number of populations that may help answer a research question, such as CD3+ cells. Finally, the data is visualized and statistically analyzed, for phenotype identification, population correlation, cell percentage, and population and CFU correlation.

The workflow for the analysis pipeline includes reading flow cytometry data output, cleaning and feature engineering this data, performing data visualization, and performing hypothesis testing through integrating other experimental measurements (Fig. 1). Each step is described in detail below, and a route map describing the method can be found in Supplementary Fig. S1.

### Cleaning data with gating input

Flow cytometers use a standardized file format for outputting data, the .fcs file, which includes cell measurements, metadata describing data collection, and the Median Fluorescent Intensities (MFIs) of fluorescently-conjugated antibodies or fluorescent probes ^8^. Multiple .fcs files generated from an experiment can be read into R and manipulated as a “flowSet” object^9^. Our pipeline begins by reading experimental data into a “flowSet” object, then cleaning the data using the *openCyto* package (Fig. 1). This package provides infrastructure for the use of reproducible algorithms to gate cells based on marker density^3^. However, it alone is unable to control for instances where clumps of cells pass through the flow cytometer lasers, producing erroneous results and subsequently skewing the data. To address this phenomenon, the “singletGate” function from the *flowStats* package is used to remove doublet or larger cell clumps^10^. The pipeline then funnels the data through the “mindensity” function, selecting for leukocytes via a threshold filter that distinguishes between populations based on cell density^3^. Finally, a “mindensity” gate is used with a live/dead stain (Zombie NIR), to filter the data to only live cells^3^. The data is next converted from a “flowSet” object into a dataframe object that complies with the “tidy data” standards, allowing further pipeline steps to draw on the powerful suite of “tidyverse” tools in R^11^.

### Feature engineering using FMOs

FMOs are often used in manual gating to control for data spread and spillover events, which are common during flow cytometry data collection^12^. Take for example a panel consisting of 10 markers with different fluorophores. When excited, each of those 10 markers fluoresce at different intensities along the light spectrum. However, while they have different spectrums, tails of these spectrums can overlap. This overlap can lead to noise within a parameter’s measurements, and in extreme cases, to the detection of false positives/negatives in the presence or absence of a marker. FMOs are created experimentally; by running parallel samples where each sample has just one marker removed from the overall panel, all cells are guaranteed to be truly negative on that marker. With FMOs, we can therefore identify a threshold for the maximum parameter values possible for true negative marker signal on cells to determine marker presence in fully stained samples^12^. Incorporation of FMOs greatly reduces the subjectivity of manual gating and helps support unbiased analysis of flow cytometry data. Despite the importance of FMOs for accurate analysis, limited flow cytometry computational tools exist that incorporate them into unsupervised analysis^13^.

**Figure 2:**
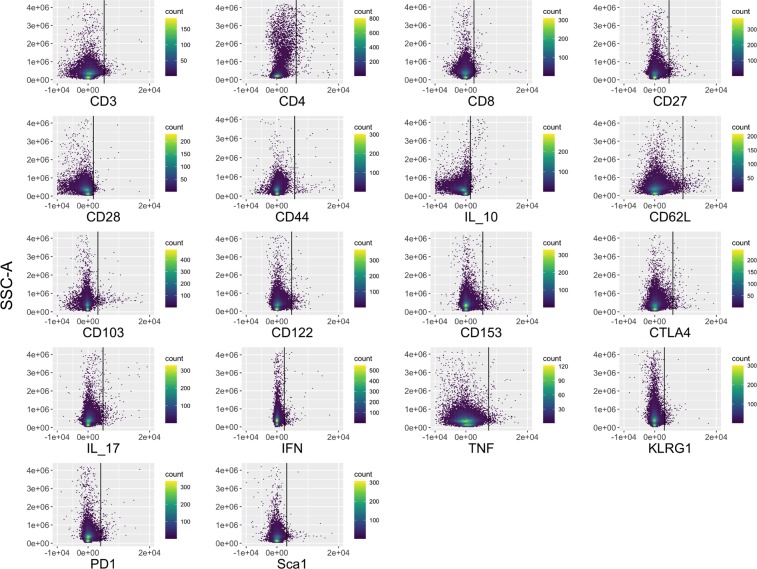
Numerical feature engineering of FMOs. The 18 FMOs are shown with the individual marker MFI expression on the x-axis and Side Scatter (SSC-A) on the y-axis. The black vertical line indicates the 99^th^ percentile threshold for identifying positive versus negative cells (i.e., 99% of the data is located to the left of the line in each plot). These thresholds are used on the subsequent samples to feature engineer new parameters on whether a cell positively or negatively expresses each marker.

Our pipeline processes the data from FMOs to include in further analysis. Traditionally, FMOs have been manually gated to identify the upper threshold of a parameter’s value for negative cells. In our pipeline, we instead automate this analysis of the FMOs, measuring the threshold as the 99^th^ percentile of the parameter values in each FMO (Fig. 2). Noise can originate from very small particles or debris that pass through the flow cytometer. In an ideal world, a 100% threshold could be used, but in reality, the 99% threshold is used to account for this random noise. The user can assess this 99% threshold with the FMO plots in Fig. 2 and adjust the thresholds if need be. The 99th percentile values are then saved and subsequently funneled into feature engineering of binary features (negative/positive) for each marker in the experimental data.

### Feature engineering identifies all cell phenotypes present in the samples

Features are measurements in a dataset, such as the MFIs used in flow cytometry. Feature engineering is a machine learning technique that uses the original features in a dataset, possibly with the integration of external knowledge or data, to create new features that make the data easier to understand^14,15^.

For flow cytometry, FMOs can add information about the possible range of expression measurements for cells that are truly negative for a marker. The threshold identified by FMOs can be used to create new binary features that capture whether the expression of each marker is positive or negative for the cell, thus, simplifying overly redundant, continuous MFI data with noise resulting from spillover. In the pipeline, we feature engineer each parameter using the thresholds identified from the FMOs, so that positive expression on cells (values above the FMO cutoff) equal 1 and negative cells equal 0.

**Figure 3:**
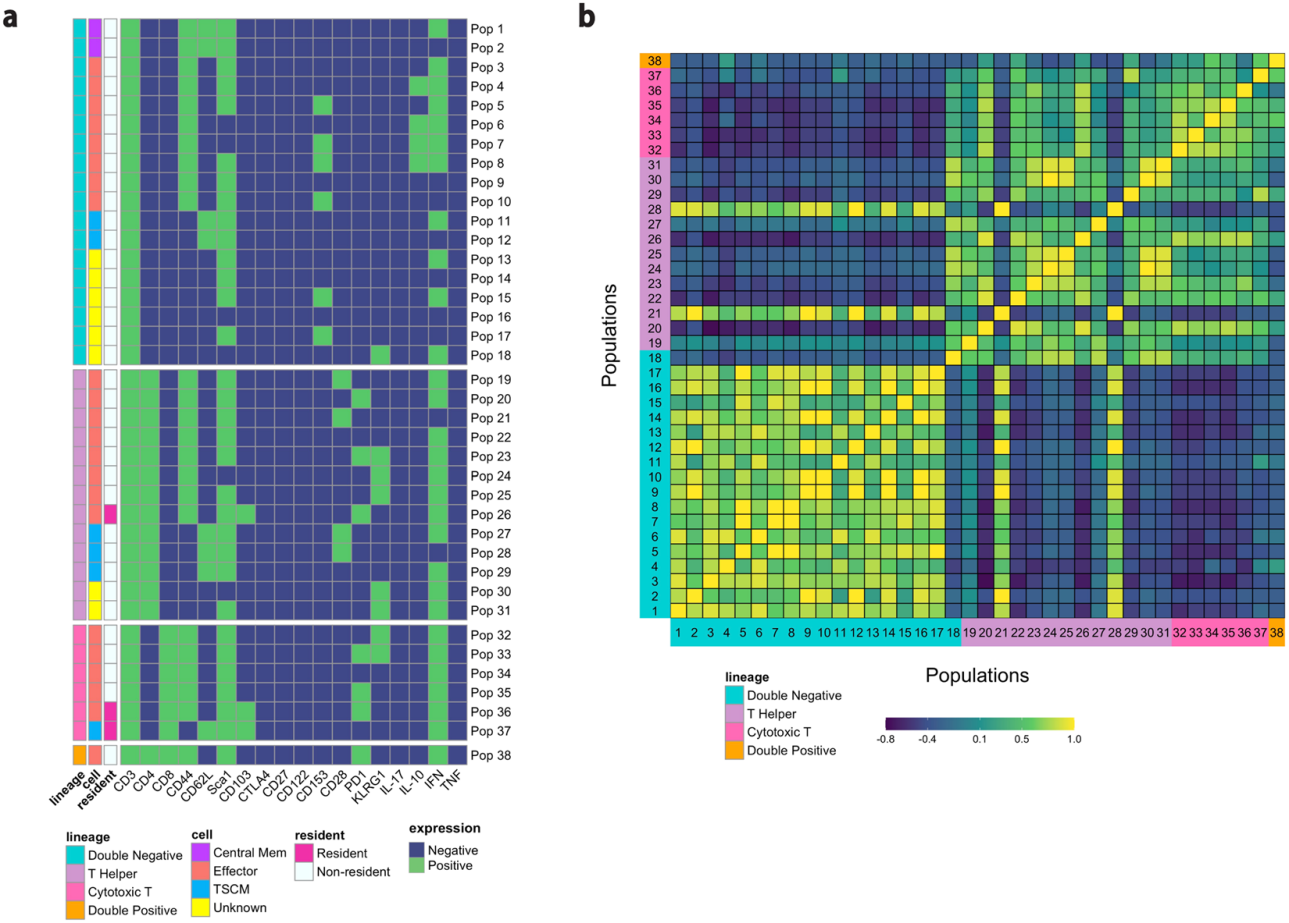
Feature engineering identifies CD3+ phenotypes and correlates identified populations. (**a**) The heatmaps show the CD3+ phenotypes that constitute greater than 0.5% of the live leukocytes in at least one sample. Green indicates positive expression, and blue indicates negative expression of all 18 markers used for analysis in the flow cytometry experiment on the x-axis. The plots are separated by four different CD3+ lineages based on CD4 and CD8 expression (double negative immune cells, T helper cells, cytotoxic T cells, and double positive T cells). The “cell” column classifies cells as either central memory, effector, T stem-cell like memory, or unknown cells that need to be explored further. The “resident” column indicates if the population is a resident cell, as determined by expression of CD103. (**b**) The correlation across study samples between the percentage of cells in each population can be used to see the similarities and differences between different populations. Yellow indicates high positive correlation and purple is high negative correlation. Populations are grouped by cell lineages, and each number on the x-axis and y-axis identifies a separate cell population, corresponding to the population numbers in Fig. 3a.

For each cell in the experimental data, the cell phenotype is then identified based on the set of marker expressions (0’s and 1’s) of each population. Eighteen markers were used to elucidate memory T cell populations including markers for terminal differentiation and exhaustion in the *M. tuberculosis* case study. The pipeline identifies all cell populations (i.e., combinations of negative and positive marker expression values) for which at least one sample includes at least one cell. A total of 12,122 cell populations were identified in the samples for this study (Supplementary Fig. S2). As this number of populations is still very large, the data can be filtered to look at a smaller subset of the populations. In this case, we are specifically looking for CD3+ T cells that may mediate protection against *M. tuberculosis* infection. Immunologically, a protective population is unlikely to be present only in extremely small numbers. Therefore, in the filtering step of our pipeline we chose to filter to CD3+ T cell populations with population percentages greater than 0.5% in at least one sample (Fig. 3a). This analysis filtered the cells to look specifically at larger populations, but an alternative filter could be used to look at rare populations that compose <0.1% of the sample, for example.

The pipeline then classifies specific lineages and subsets of cells according to marker expression (Supplementary Tables S1 and S2)^16,17^. The pipeline identifies cell phenotypes that may be overlooked in manual analysis, due to combinations of markers that are uncommon (e.g., cells classified as “unknown” in this analysis) or combinations that have not yet been linked to a disease or condition of interest. In the case study, for example, there are six populations that express CD153; CD153 has only recently been shown to mediate protection against *M. tuberculosis*^18^. These six double-negative populations likely would not have been gated for in a manual gating scheme, as thus far, the marker has only been shown to be present on CD4+ T helper cells^18^.

While we used T cell lineages and subsets in the case study analysis, the pipeline could easily be modified for different panels. If a panel aims to identify myeloid cells, they could be classified by lineage as macrophages, neutrophils, dendritic cells, and then subset further, for example, by alveolar macrophages and interstitial macrophages.

### Correlation between identified populations

The pipeline then visualizes a correlation matrix comparing the percentage of cells in each of the populations (Fig. 3b). This allows users to explore associations between cell populations. This plot can also allow for identifying unusual populations that do not behave like other populations within the same lineage. In the case study example, populations 21 and 28, which are T helper cell populations, are negatively correlated or uncorrelated with the other T helper cells (populations 19–31), and instead have patterns more similar to the identified double-negative cell populations. These are the only identified cell populations outside the double-negative cells that are negative for interferon-gamma (IFN-γ) expression. Importantly, IFN-γ is well known to be strongly associated with protection against *M. tuberculosis*^19^. As these cell populations do not behave similarly to the other T helper cells, they may be a good candidate for further, more targeted exploration in later experiments.

### Visualizing the percentage of cells in each population

**Figure 4:**
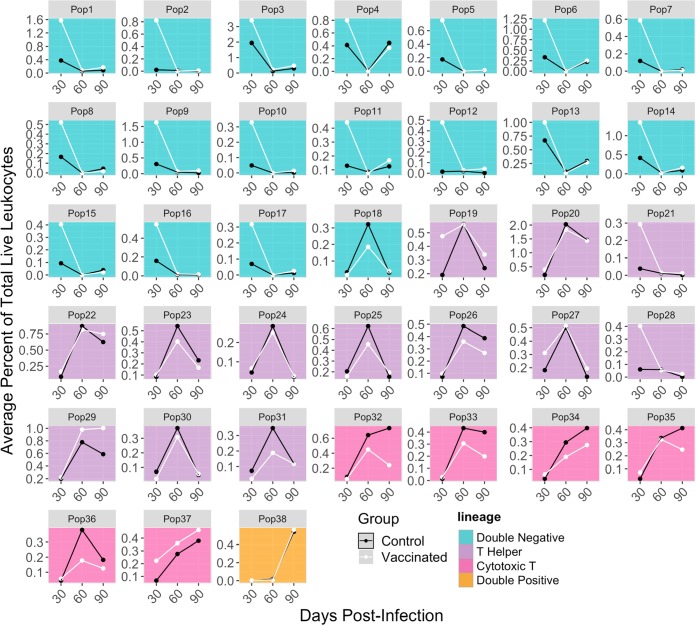
Time series of percentage of cells in each population. Each small plot shows the time series of a single cell population identified in the pipeline at the three measured timepoints post-infection. Separate lines are shown for vaccinated (“BCG”) versus control mice. Each point represents average cell populations across all mouse replicates (4–5 per time point and vaccination status). The small plots give cell populations in the same order as Figs. 3 and 5, with population identification numbers corresponding across the three plots. The background color of each plot matches the cell lineages plotted in Fig. 3a.

Whereas less modular pipelines may provide more limited options for visualizations at later stages of analysis, our pipeline’s modularity and its use of a common “tidy” data format provide the researcher wide flexibility to create visualizations suited to their research question and data characteristics through the *ggplot2* package^11^. For this study, the percentage of cells in each population at each timepoint is plotted to compare the dynamic changes in populations over time and between groups (Fig. 4). Some populations may steadily increase or decrease over time, while others behave in unexpected ways. Comparing the two groups, we can see that some cell populations (e.g., population 20) are similar over time regardless of vaccination status. It is also possible to elucidate differences based on cell lineages. For example, the largest differences in population percentages occurs at Day 30 post-infection, before BCG-vaccination protection begins to wane^20^. This difference primarily occurs in the double-negative CD3^+^ immune cell populations. This example visualization, and its accompanying code, is just one application of visualizations that could be created at this step of the workflow (see Supplementary Fig. S3 for two more examples).

### Integrating cell population measurements with other experimental measurements

**Figure 5:**
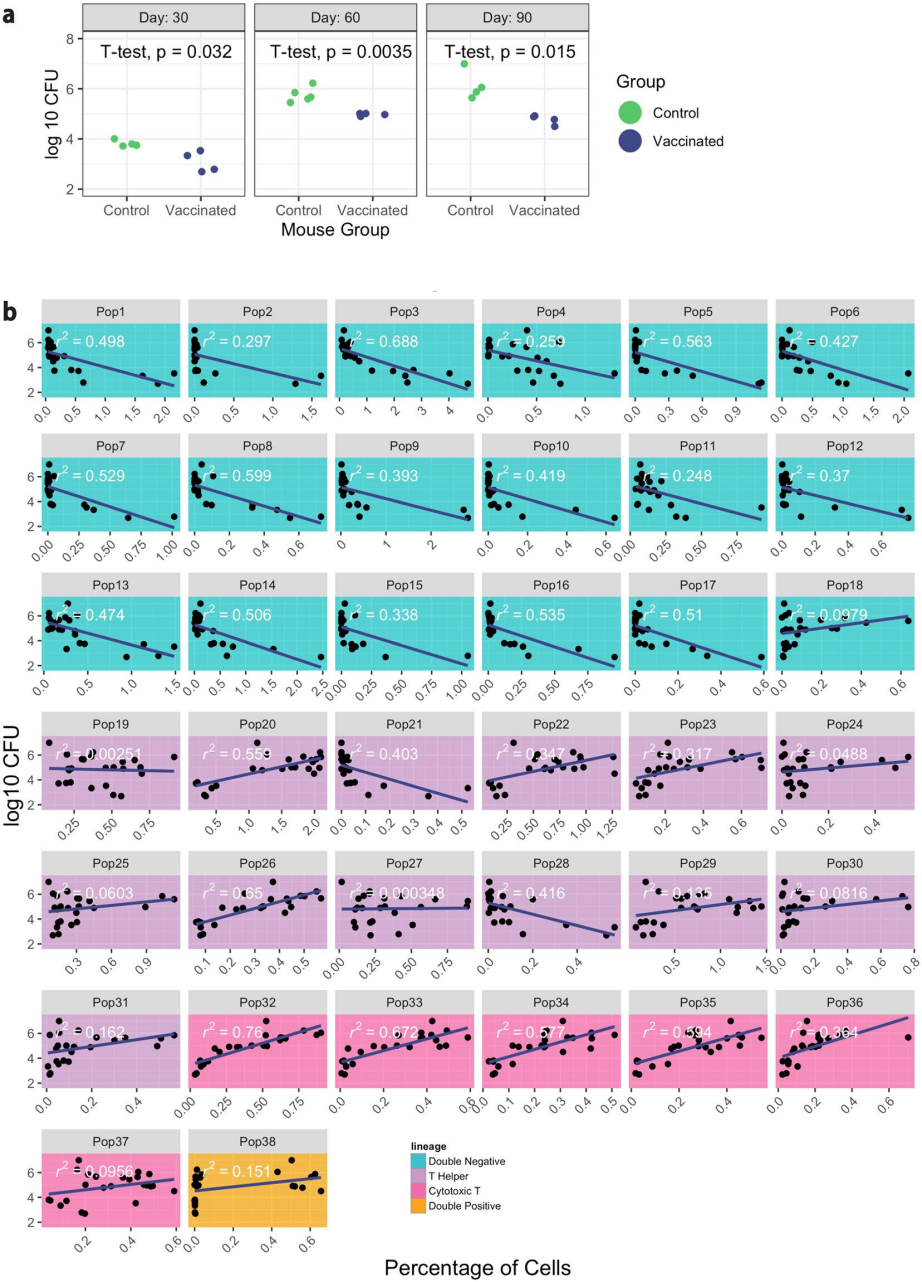
Populations associated with bacterial burden. (**a**) The log_10_^−^ *M. tuberculosis* CFUs in each mouse at each time point is shown, separated into vaccinated (“BCG”) and control groups. Unpaired t-tests were used to calculate statistical significance (p < 0.05). (**b**) Each small plot shows the association between a specific cell population and bacterial burden across all samples for the experiment. The small plots give cell populations in the same order as Figs. 3 and 4, with population identification numbers and lineage colors corresponding across the three plots. The x-axis in each small plot gives the percentage of cells in a population, with each point providing the measurement from a single mouse. The y-axis gives the log_10_ *M. tuberculosis* CFUs for that mouse. Estimates of how well the linear models fit the data between cell population sizes and log_10_ CFUs are given on each plot (“r^2^”).

At this stage, the pipeline allows the integration of cell population measurements with other data from the experiment, such as lesion scores or gene expression. In the *M. tuberculosis* study, bacterial burden (expressed as log_10_ transformed Colony Forming Units (CFUs)) is a measurement of the number of bacteria found in the lung. These CFU measurements were found to vary between experimental groups in the case study data, with significantly higher bacterial burden at days 30, 60, and 90 post-infection in the control group compared to the vaccinated group (Fig. 5a). It is of interest to investigate if certain cell populations identified through the pipeline, are associated with this measurement of bacterial burden, as this might help to identify cell populations possibly indicative of the host’s response to infection with or without vaccination.

For this case study, the pipeline investigates associations between CFUs and cell populations using scatterplot visualizations and linear regression (Fig. 5b). It tests the null hypothesis of a slope of zero for CFUs regressed on cell population size within a mouse’s lung (Fig. 5b and Supplementary Table S3). Further, the coefficient of determination (r^2^) was estimated between the CFUs and each cell population. This analysis identified that cells that co-express CD44, CD153, and IFN-γ (populations 5, 7, 8) could be candidates for future experimental studies. They are potentially protective against *M. tuberculosis* infection, as a decreased bacterial burden is associated with a higher percentage of these populations.

In this section of the pipeline, the user can modify the statistical model and visualization used in the case study for a wide range of alternatives. For example, the linear model fit in this step of the example pipeline is based on an assumption of linearity in the relationship between cell populations and CFUs, but exploratory analysis might identify that this assumption is incorrect. In this case, since the pipeline is modular, the code fitting the linear models can be replaced with R code to fit non-linear or non-parametric models. Further, the pipeline allows users to add analysis steps at this point. For example, when performing multiple statistical tests there is an increased possibility of identifying false statistically relevant comparisons^21^. The user might want to adjust the acceptable p-values for the multiple comparisons made in fitting linear models for each cell population. The pipeline could be extended with the Benjamini and Hochberg False Discovery Rate correction (e.g., Supplementary Table S3) or other multiple testing corrections (e.g., Bonferroni or Benjamini & Yekutieli)^22–24^.

### Population validation via manual gating

As with the development of any new tool to analyze data, the pipeline must be tested against a traditional method, in this case, manual gating, to ensure that similar patterns and populations are captured in both analyses. To investigate if the estimated cell population sizes were similar between the automated and manual gating, a population for which we observed a relatively high percentage of cells (population 3) was manually gated (Supplementary Fig. S4). The percentage of cells in each of the populations at the different time points are very similar to the percentages calculated in this analysis pipeline with an average absolute difference of 0.26%. There is also a high correlation coefficient (ρ_rs_ = 0.99, p < 0.01), indicating a strong positive association between the manual and pipeline gating (Supplementary Fig. S4).

### Running time

Measurements for running time were made on a computer with 32GB RAM and a 4.2 GHz Intel Quad Core i7 processor. The initial data files contain a total of 7,299,424 cells (1.24GB); the FMO files contain 2,641,651 cells (0.57GB). Following gating, there were 468,754 cells from the FMOS and 1,023,402 cells from the sample data. These 1 million data points were input to the feature engineering algorithm. From the gating steps and feature engineering, through producing the plots in Fig. 3a and Supplementary Fig. S2, the analysis script took 2.7 minutes running time. Half of this was spent on the gating steps (1.01 min). The running time was also evaluated based on the number of input cells to the feature engineering algorithm (Supplementary Fig. S5). It analyzes roughly 10,000 cells per second.

### Testing the pipeline with clinical human whole blood

**Figure 6:**
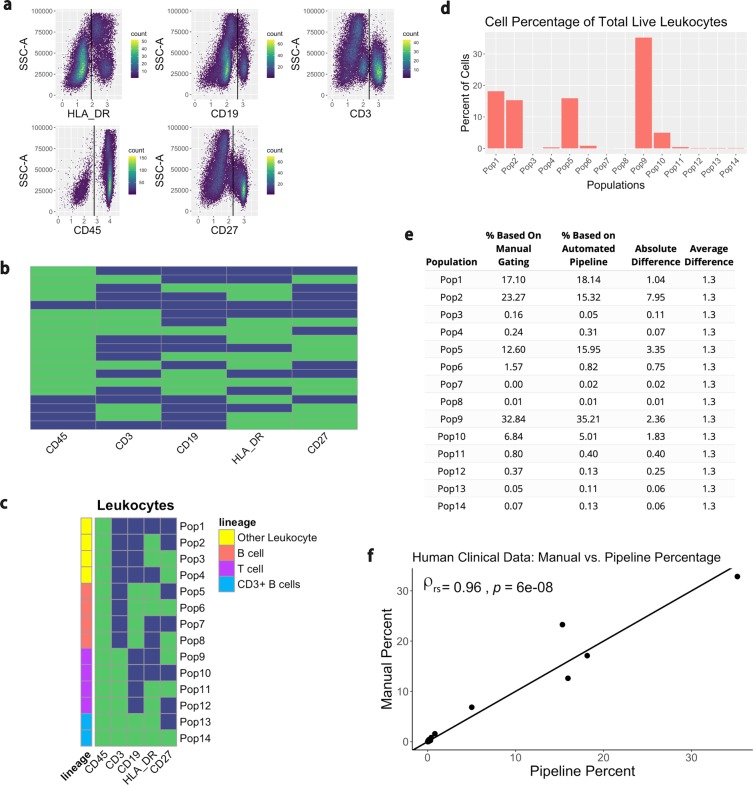
Testing the pipeline with human clinical samples. The data is first transformed so that it is viewable on a linear axis. (**a**) A “mindensity” gate from the R package *openCyto* is then used to determine the cutoff for positive and negative MFIs. (**b**) The data is feature engineered based on these cutoffs and identifies a total of 19 different populations. (**c**) The populations are then filtered to only view the leukocytes, or CD45+ populations. These populations are further subset by lineage. (**d**) The percentage of cells in each population is displayed. (**e**,**f**) Each population was manually gated in FlowJo and the difference in the percentage of cells from manual gating and the automated pipeline was compared. A table and plot compare the differences for each of the populations. Each point on the plot indicates a measurement for one of the 14 populations identified. The x-axis represents the value for the percentage of cells found via the automated pipeline, while the y-axis represents the percentage of cells found via manual gating. The y = x line through the center of the plot indicates where the points would be located if both of the gating methods output identical values. The Spearman correlation coefficient (ρ_rs_) comparing the methods and the p value are displayed at the top left of the plot.

Another dataset analyzing clinical human whole blood in a healthy individual was used to confirm the utility of this pipeline. This data was collected and compensated on a Fortessa II with a panel comprised of 5 markers: CD45, CD3, CD19, HLA-DR, and CD27. When used side-by-side, FMOs are a more robust depiction of the fluorescent marker composition in a sample as they account for spillover from other channels. However, it is not always possible to run FMOs. When there is good separation between the positive and negative populations within a marker, it is acceptable to base the MFI cutoffs on the sample data^12^. The “mindensity” function from the *openCyto* package was used on the sample to determine thresholds for the feature engineering (Fig. 6a). Following feature engineering, a total of 19 populations were identified in the sample (Fig. 6b). The CD45+ populations, or leukocytes, were filtered and subset according to B cell and T cell lineages (Fig. 6c). The percentage of cells in each of the populations is shown in Fig. 6d. Finally, the comparison of the percentage of cells between the manual gating and the automated pipeline indicated high similarity between the two methods (Fig. 6e,f). The Spearman correlation coefficient (ρ_rs_) between the two methods is 0.96 with a p-value < 0.001. There is therefore slightly more discrepancy between automated and manual gating for this dataset compared to the murine lung cells, although the ranking agreement between the two methods is still very high. The use of this clinical dataset shows the utility and flexibility of the pipeline for conventional flow cytometry without the need for FMOs.

## Discussion

The exploration of large and complex biological datasets requires steps of simplification and aggregation; scientific research is improved if these steps draw on biological knowledge and principles, rather than relying exclusively on subject-agnostic statistical techniques. Flow cytometers generate data with continuous measurements of an underlying binary phenomenon, with much of the added noise in measurements due to spillover from other channels. FMO controls can be used to informatively distinguish negative and positive cell populations for a marker in flow cytometry data and are used for this purpose commonly in traditional manual gating. However, they have not yet been integrated with the open-source tools being created to improve objectivity and efficiency of flow cytometry analysis. As with any simplification step, the pipeline presented here does lose some of the information inherent in the original measurements. In cells that are positive for a marker, for example, the measurement of expression density may have some biological meaning, which is lost in creating binary distinctions in the feature engineering. Future development of this pipeline could focus on extending the methods to help explore meaningful variation among the positive values for a marker. In the case of markers in which there is low, intermediate, and high expression, the 99th percentile FMO cutoff could be used to determine the negative population. From here, a function like “mindensity” used to analyze the clinical data could be used to determine the minimum density between the intermediate and high population, thus splitting the marker into three expression levels. If a more continuous expression level is of interest, instead of using the “mindensity” function to separate the positive markers into intermediate and high expression, the continuous marker expression could be retained.

This pipeline’s modularity adheres to the Unix philosophy of combining small tools that each perform a discrete task to solve complex problems^25^. Excellent tools already exist in the R ecosystem, both specific to flow cytometry data (e.g., to perform initial gating on samples) and for more general data visualization and modeling. Rather than developing a “fixed” pipeline, where pre-existing tools are encapsulated within a framework that does not allow easy modifications or substitutions of steps in the pipeline, this pipeline is based on the principle of combining these existing tools from the R ecosystem. We designed this pipeline to use a common data format in later steps, which makes it useful to a variety of researchers, as it is easy to adapt with common R tools. For example, in the visualization and statistical testing stages, different statistical models and plots can easily be substituted for the linear regression that is used for the data described here. Another advantage of this modular pipeline is that it can be used on clinical and research cytometry samples from either conventional or spectral flow cytometers.

An added strength of the pipeline is that, since it is computationally simple, it does not require down-sampling of the raw flow cytometry data. For clustering algorithms that are computationally intensive (e.g., t-SNE), it is often necessary to down-sample data by taking a random sample of the data in order to improve computation time^26,27^. This manipulation of data can lead to the masking of novel discoveries. Each sample has a different total live leukocyte cell count allowing us to consider percentage of cells rather than total cell count throughout the pipeline. As our goal is to identify immune cells that are biologically involved in the protection against disease, we filtered the cell populations to those comprising >0.5% of cells in at least one sample. In this case, even if only one animal in a group has a high percentage of cells in a certain population, it will be maintained to compare against all groups. In other contexts, for example, in cancer research where the goal is to identify very rare cell types, this percentage could be decreased with a low-set filter (e.g. to maintain populations with <5 cells). The filtering and the feature engineering analyze the data in a way that makes it immediately interpretable to immunologists without the hassle and bias of manually looking for populations of interest or trying to understand complex clusters from other methods.

Another advantage of the pipeline is its ability to compile additional flow cytometry datasets. Other multiparametric approaches, such as UMAP and t-SNE, typically assign cells to unnamed clusters^5,28^. To compile replicated data, these methods map additional data back to the original data. Unlike these approaches, cyto-feature engineering does not assign data to unnamed clusters, but rather keeps groupings explicitly tied to specific, named markers in the original data. Provided that replicated or additional data contains the same markers as the original data, it can be seamlessly added to the pipeline by adding the data to the data folder. Any additional cell populations identified will be added to the plots when the analysis is run. Further, the data with cyto-feature engineering does not need to be manually gated to determine statistical significance between populations. Statistical analysis is a built-in feature that can be calculated based on the identified populations.

Researchers have an inherent bias in the types of cells they gate when analyzing flow cytometry data. By identifying all phenotypes present in the dataset, this pipeline allows users to analyze a variety of populations in an unbiased manner. These cell types, such as the CD153+ cells in the murine lung data, may have gone undiscovered if not for the use of this fast and reproducible pipeline. Importantly, this analysis pipeline relies on high quality flow cytometry methodology, and/or FMO samples, as well as, strong panel design. Spillover from other channels can greatly impact the analysis, so researchers must ensure that the controls are prepared correctly.

The analysis pipeline described here, allows for the use of necessary and rigorous technical controls in flow cytometry. The pipeline is able to identify populations that may not be normally gated. Provided that the samples and controls remain constant, the automated pipeline analysis will consistently produce the same results, removing person-to-person bias. Further, this pipeline drastically reduces the amount of time typically required to analyze flow cytometry data. Overall, this strategy is envisioned to help identify the elusive nature of cellular phenotypes through fast and accurate analysis of flow cytometry data.

## Methods

### Experimental setup

The experiment was designed to compare T cell populations between BCG-vaccinated and control (PBS-injected) mice following infection with *M. tuberculosis*. The populations could then be compared to the bacterial load (CFUs) to associate the immune cells with disease.

### Animals

C57BL/6 mice were purchased from the Jackson Laboratory (Bar Harbor, ME). The mice were retained in a BSL-3 facility at Colorado State University, and all experimental protocols were approved by the Institutional Animal Care and Use Committee at Colorado State University. All methods were carried out in accordance with relevant guidelines and regulations for care and use of laboratory animals.

### Vaccinations

Mice were vaccinated subcutaneously with 1 × 10^6^ CFU *M. bovis* BCG Pasteur or 100 µL of phosphate buffered saline (PBS) (Corning, Corning, NY).

### Mycobacterium tuberculosis Infection

Twelve weeks after vaccination, mice were aerosol challenged with *M. tuberculosis* HN878 grown in 7H9 broth and stored at −80 °C. The *M. tuberculosis* was suspended in PBS and aerosolized using an aerosol chamber (Glas-Col, Terre Haute, IN). Aerosolization delivered 164 CFU/animal confirmed via plating whole lung homogenates on the same day of infection.

### Tissue preparation

Mice were euthanized by CO_2_ inhalation. The superior and middle lung lobes were harvested and digested with DNase I- type IV Bovine (2.6 units/ml; Sigma-Aldrich, St. Louis, MO)/ Liberase (750 units/mL; Sigma-Aldrich, St. Louis, MO) at 37 °C for 30 minutes. The lungs were strained through a 70 µm cell strainer and treated for 1 minute with Red Blood Cell Lysing Buffer (Sigma-Aldrich, St. Louis, MO) to lyse erythrocytes. Complete DMEM, composed of 500 mL of 1x DMEM (Corning, Corning, NY), 45 mL of fetal bovine serum (Atlas Biologicals, Fort Collins, CO), 4.5 mL MEM non-essential amino acids (Corning, Corning, NY), 4.5 mL of Penicillin Streptomycin (10,000 units/mL Penicillin, 10,000 µg/mL Streptomycin; Thermo Fisher Scientific, Waltham, MA), and 4.5 mL of L-glutamine (Sigma-Aldrich, St. Louis, MO), was then added to neutralize the solution. Lung cells were resuspended in 500 µL of PBS (Corning, Corning, NY).

### Flow cytometry and analysis

Following single-cell suspension of lung cells in PBS, cells were stimulated with 1x BD Golgi Stop, a protein transport inhibitor (BD Biosciences, Cat# 554724) in complete media for 3 hours in a 37 °C incubator. Cells were then washed with PBS and incubated with a 1:2,000 dilution of Zombie-NIR viability stain (BioLegend, San Diego, CA) for 15 minutes in the dark at room temperature. After a wash with FACS Staining buffer (PBS (Corning, Corning, NY) containing 2% Fetal Bovine Serum (Atlas Biologicals, Fort Collins, CO) and 0.05% sodium azide (Thermo Fisher Scientific, Waltham, MA)), cells were incubated for 30 minutes at 4 °C with a fluorescently-labeled surface antibody cocktail containing a 1:200 dilution of FC Block (See Supplementary Table S4). Following a wash with FACS staining buffer, cells were incubated with 150 µL of 1x Permeabilization/Fixation buffer (Invitrogen, Carlsbad, CA) for 1 hour at room temperature. Cells were subsequently washed with 150 µL of 1x permeabilization buffer (Invitrogen, Carlsbad, CA) before being incubated with an intracellular cytokine antibody cocktail overnight at 4 °C in the dark. Cells were then washed and finally resuspended in 1x permeabilization buffer. Eighteen markers were used to analyze memory T cell expression: Sca-1, CD3, CD62L, CD122, CD28, PD-1, CD103, CD44, CD4, CD8, CTLA-4, CD27, CD153, KLRG-1, IL-17, IFN-γ, IL-10, and TNF-α (Supplementary Table S4). Antibodies and reagents were purchased from BD Biosciences, BioLegend, or Thermo Fisher Scientific. 100,000 events were collected per sample on a Cytek Aurora Flow Cytometer (Cytek, Fremont, CA) and analyzed with FlowJo version 10.5.2 software and the cyto-feature engineering pipeline. Cell populations were identified by unsupervised feature engineering of cells by phenotype and confirmed via traditional gating methods.

### Bacterial burden

One third lungs were placed in a Bullet Blender Blue (Next Advance, Troy, NY) and homogenized at speed 8 for 4 minutes. Tissue homogenate was plated at a 1:5 dilution on 7H11 agar plates. The limit of detection is 15 CFUs for lungs.

### Human clinical data

Human clinical data was obtained and processed as previously described^29–31^. All experimental protocols involving human subjects were approved by the Ethics Committee of the Institute of Medical Research of the Faculty of Medicine at the University of Antioquia and adhere to the ethical principles outlined in the Declaration of Helsinki. EDTA-anticoagulated (4 mL) whole cells were obtained from peripheral blood of healthy volunteers aged 20–30 years, after written informed consent to participate. Most of them worked in the Sede de Investigación Universitaria (SIU) at Universidad de Antioquia and signed a written informed consent approved by the Ethics Committee of the Institute of Medical Research of the Faculty of Medicine at the University of Antioquia. They declared that they were not taking any medication and that they had neither an autoimmune nor active infectious disease.

Thirty microliters of whole blood cells suspended in 100 µL eBioscience Flow Cytometry Staining Buffer (Cat# 00-4222-26) and stained with fluorochrome-conjugated mouse anti-human 0.5 µL CD45-PE-Cy7 (Clone: HI30), 5 µL CD3-PE (Clone: OKT3), 7 µL CD19-Alexa Fluor 488 (Clone: HIB19) and 5 µL CD27-APC (Clone: M-T271) monoclonal antibodies for twenty minutes at room temperature. Erythrocytes were lysed with 300 μL of OptiLyse Buffer for 10 minutes and 300 μL of sterile deionized water for an additional 10 minutes. The acquisition was performed in an LSR Fortessa II flow cytometer.

### Statistical analysis and reproducible code

Statistical significance for CFUs was determined using unpaired t-tests (p < 0.05) using the *ggpubr *package, R version 3.6.2. The R and package versions can be found in Supplementary Fig. S6.

## Supplementary information

Supplementary information.

## Data Availability

The datasets generated during and/or analyzed during the current study are available online. The flow cytometry data can be found at Flow Repository: https://flowrepository.org/id/RvFrMfWyY0wR3ZM3cvlsAsQPBmAOXmMUzURGm1D8V0ShqSNnH2UCrPdpttuoqNS4. All other data can be found at: https://github.com/aef1004/cyto-feature_engineering in the “data” folder.

## Code availability

Code to analyze the data and produce the graphs can be found at: https://github.com/aef1004/cyto-feature_engineering.
