# Supplementary material for: Cyto-Feature Engineering: A Pipeline for Flow Cytometry Analysis to Uncover Immune Populations and Associations with Disease
Source: Sci Rep. 2020 May 6;10:7651. doi: 10.1038/s41598-020-64516-0 (PMC7203241; doi:10.1038/s41598-020-64516-0)
Supplement: Supplementary file 1 — Supplementary information. [file 41598_2020_64516_MOESM1_ESM.pdf]

Cyto-Feature Engineering: A Pipeline for Flow Cytometry Analysis to Uncover Immune Populations and Association with Disease

Amy Fox[1], Taru S. Dutt[1], Burton Karger[1], Mauricio Rojas López[2], Andrés Obregón-Henao[1], G. Brooke Anderson[3] and Marcela Henao-Tamayo[1]*

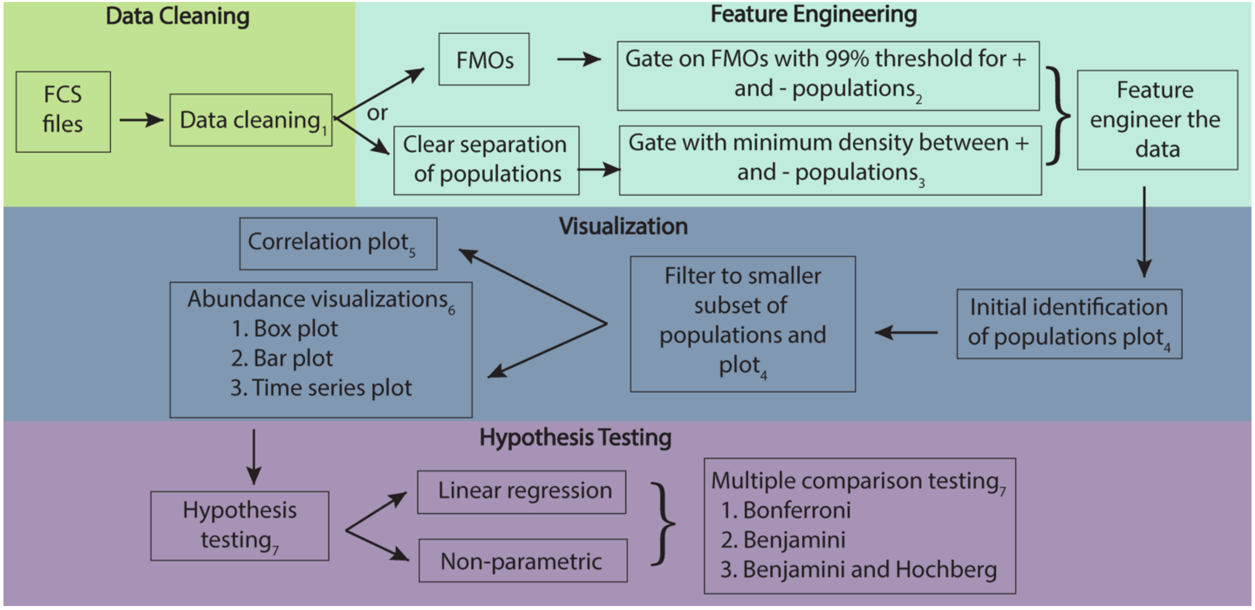

Supplementary Figure S1: Pipeline route map. The route map details the workflow of the pipeline based on the samples acquired and the visualiations and hypothesis testing of interest. The packages used at each of the stages are noted as follows: (1) *ncdfFlow*: to read in the flow cytometry data to R, *openCyto*: to facilitate automated gating for data cleaning, *ggcyto*: to visualize the initial gating strategy, *tibble*: to convert the S4 flow data object to a data frame, *flowCore*: to transform the data if needed, *dplyr*: to tidy the data (2) *quantile*: to perform the 99% FMO threshold cutoff (3) *openCyto*: to calculate the minimum density between the positive and negative cell populations (4) *pheatmap:* to plot the identified cell populations (5) *superheat*: to find correlations between different cell populations (6) *ggplot2*: to plot cell population abundances and changes in abundances over time (7) *stats*: to perform linear regressions, calculate p-values, and execute multiple comparison testing, *ggplot2*: to plot the cell abundances against other data from the experiment.

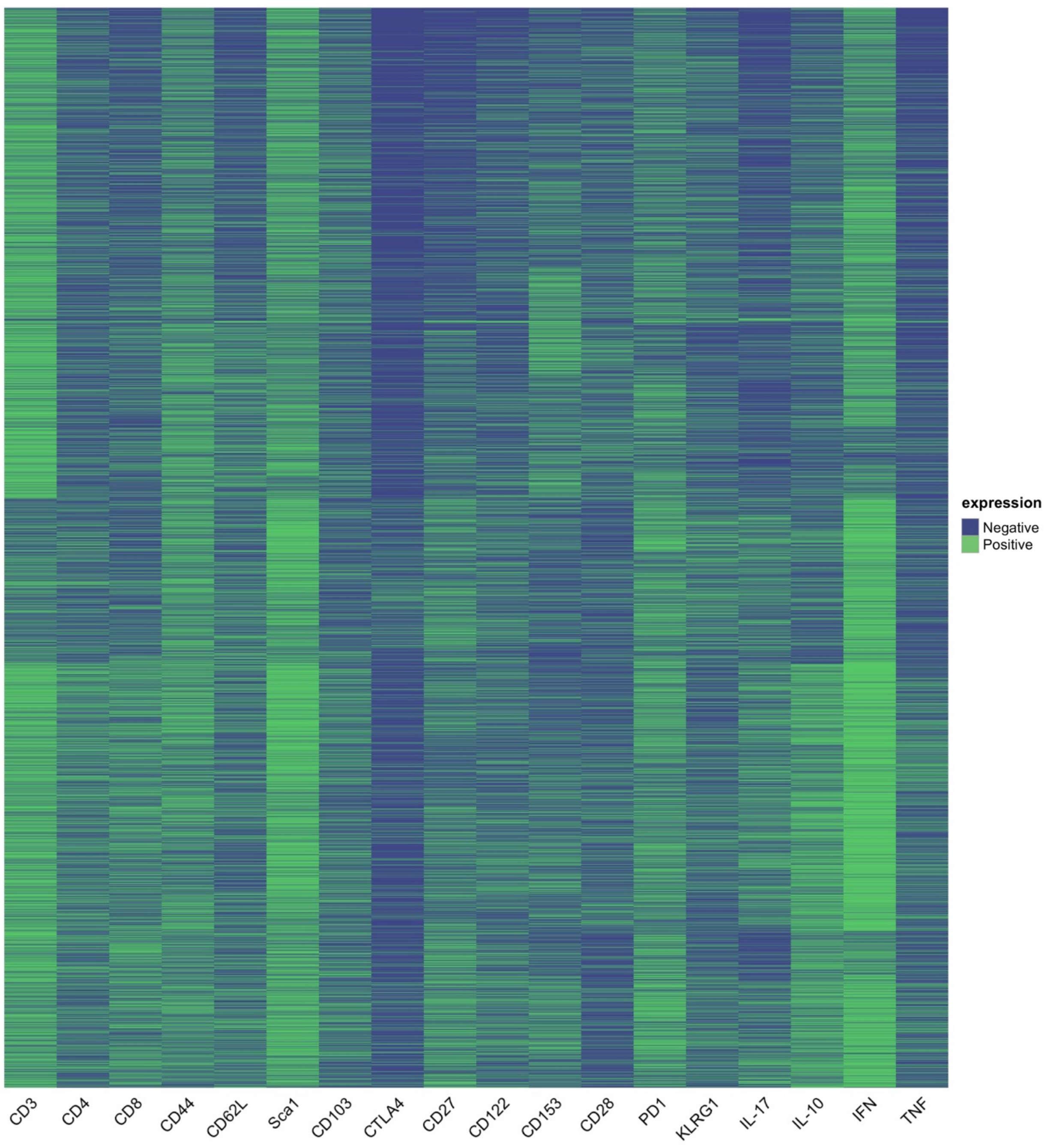

Supplementary Figure S2: Identification of all phenotypes in the lung samples. After feature engineering the data, 12,122 total populations were identified. Each row represents a unique cell phenotype, where green indicates positive expression and blue indicates negative expression of each marker.

a

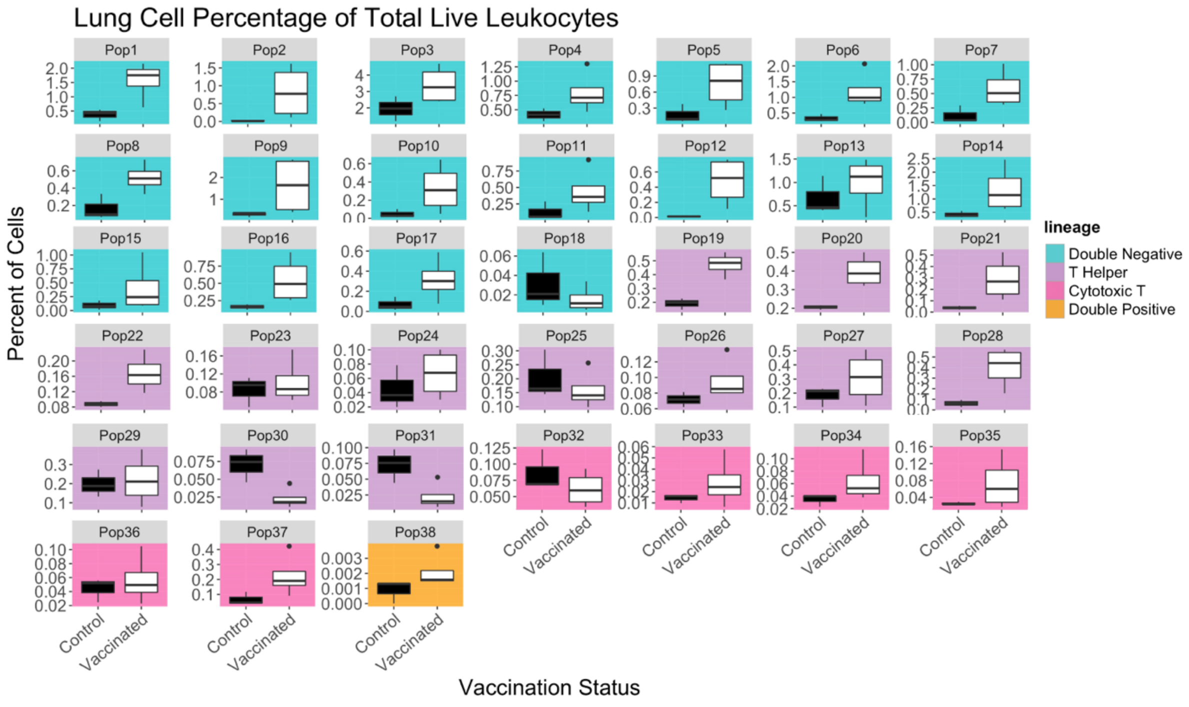

b

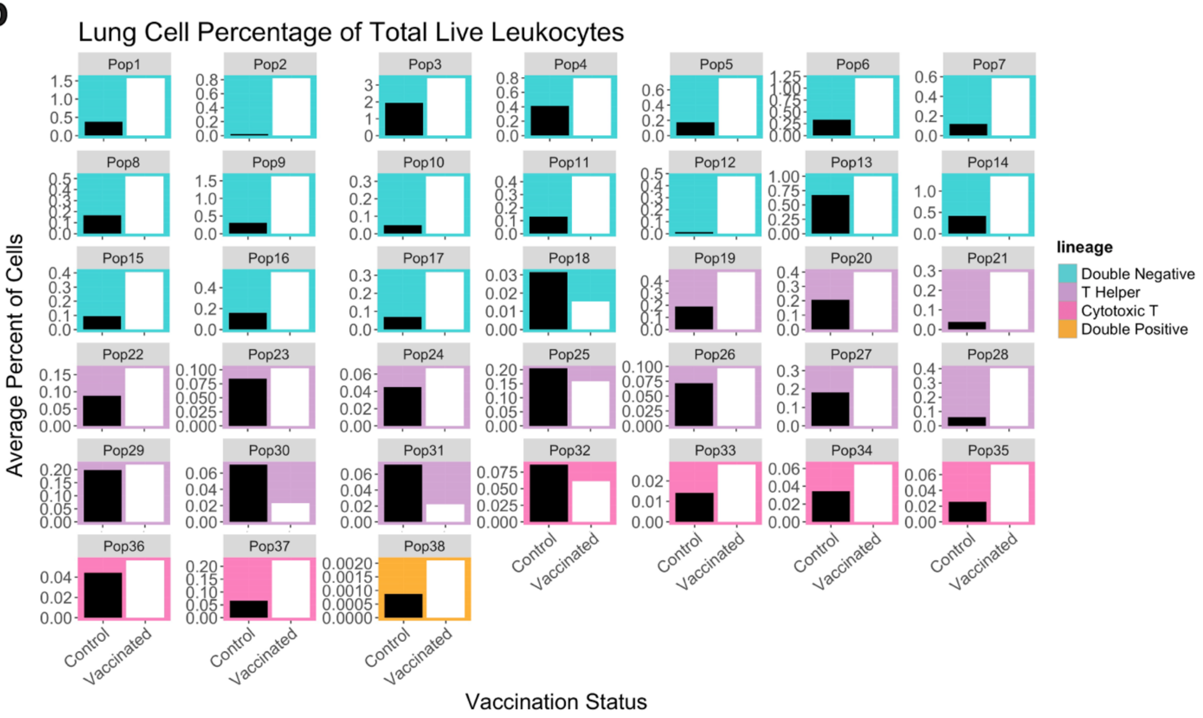

Supplementary Figure S3: Alternative visualizations for cell percentages. All of the plot backgrounds denote the cell lineages as described in Figure 3a. The day 30 data was used for all of the plots in this figure. a) Box plots show the distribution of the percentage of cells in each population. b) Bar plots show the average percentage of cells in the two experimental groups in each population.

a

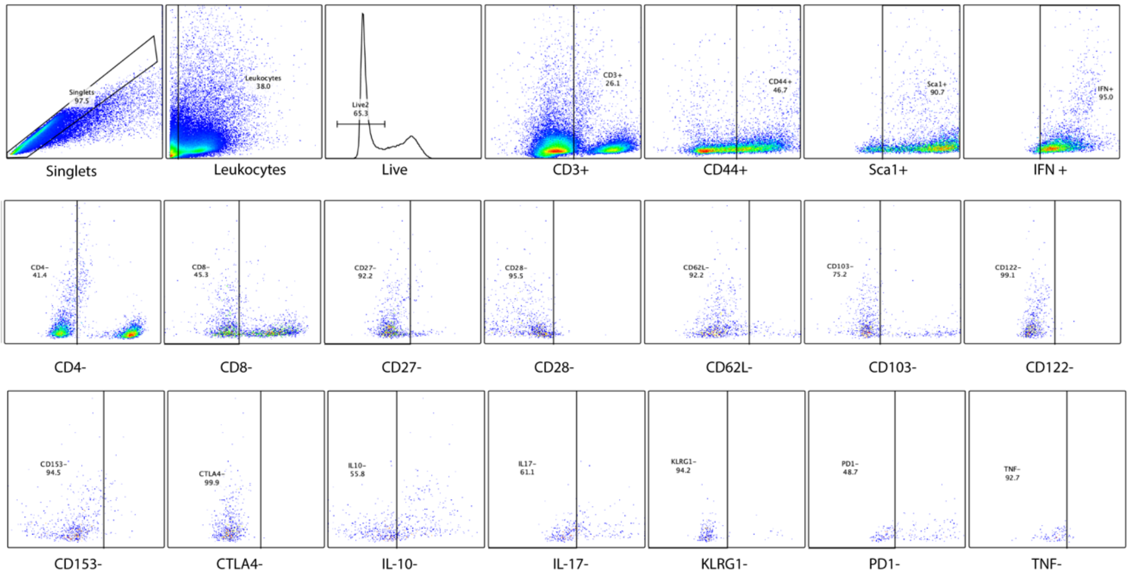

b

Timepoint	Group	Number	% Based On Manual Gating	% Based on Automated Pipeline	Absolute Difference	Average Difference
30	Control	A	2.00	2.70	0.70	0.26
30	Control	B	1.55	1.96	0.41	0.26
30	Control	C	0.90	1.19	0.29	0.26
30	Vaccinated	A	2.12	2.48	0.36	0.26
30	Vaccinated	B	3.23	4.02	0.79	0.26
30	Vaccinated	C	1.37	2.41	1.04	0.26
30	Vaccinated	D	3.34	4.69	1.36	0.26
60	Control	A	0.09	0.14	0.06	0.26
60	Control	B	0.07	0.10	0.02	0.26
60	Control	C	0.05	0.08	0.03	0.26
60	Control	D	0.07	0.13	0.06	0.26
60	Control	E	0.06	0.17	0.11	0.26
60	Vaccinated	A	0.19	0.25	0.06	0.26
60	Vaccinated	B	0.13	0.21	0.09	0.26
60	Vaccinated	C	0.15	0.23	0.08	0.26
60	Vaccinated	D	0.16	0.23	0.07	0.26
90	Control	A	0.19	0.31	0.12	0.26
90	Control	B	0.13	0.16	0.03	0.26
90	Control	C	0.23	0.31	0.09	0.26
90	Control	D	0.28	0.40	0.12	0.26
90	Vaccinated	A	0.55	0.72	0.17	0.26
90	Vaccinated	B	0.44	0.56	0.12	0.26
90	Vaccinated	C	0.39	0.40	0.01	0.26
90	Vaccinated	D	0.16	0.22	0.05	0.26

c

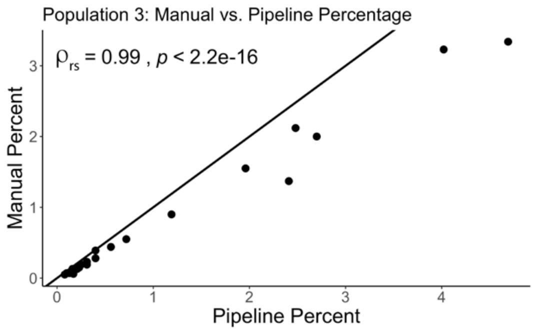

Supplementary Figure S4: Confirmation of populations via manual gating. A) Population 3, defined by CD3+ CD44+ Sca1+ IFN-γ+ CD8- CD27- CD28- CD62L- CD69- CD103- CD122- CD153- CTLA4- IL10- IL17- KLRG1- PD1- TNF-α, was manually gated in FlowJo using the FMOs. B) The difference in the percentage of cells in population 3 for each mouse at each timepoint was calculated, as well as the absolute average difference. C) A comparison of manual and pipeline gating results for this population. Each point represents the measurements of population 3 in a single mouse. The points' position on the x-axis gives the population 3 measurement based on the pipeline while the position on the y-axis gives the population based on manual gating. The diagonal line provides a reference of x = y (i.e., where points would fall if results from manual and pipeline gating were identical). The Spearman correlation coefficient (ρ_{rs}) and p value are displayed on the plot.

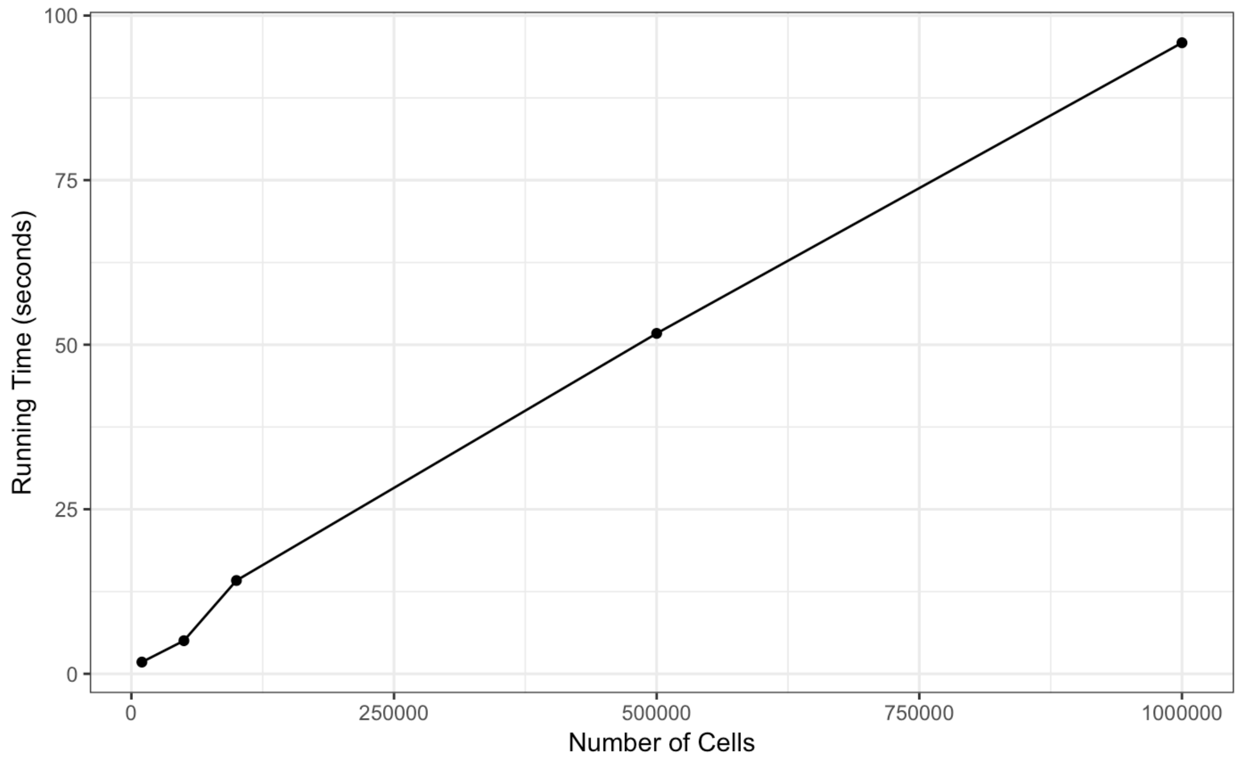

Supplementary Figure S5: Linear running time for feature engineering algorithm. The x-axis displays the number of cells that are put into the feature engineering algorithm and the y-axis displays the amount of time in seconds that it takes to compute the feature engineering using the input number of cells.

```
R version 3.6.2 (2019-12-12)
Platform: x86_64-apple-darwin15.6.0 (64-bit)
Running under: macOS Catalina 10.15.3

Matrix products: default
BLAS:   /System/Library/Frameworks/Accelerate.framework/Versions/A/Frameworks/vecLib.framework/Versions/A/libBLAS.dylib
LAPACK: /Library/Frameworks/R.framework/Versions/3.6/Resources/lib/libRlapack.dylib

locale:
[1] en_US.UTF-8/en_US.UTF-8/en_US.UTF-8/C/en_US.UTF-8/en_US.UTF-8

attached base packages:
[1] grid      parallel  stats     graphics  grDevices utils     datasets  methods   base

other attached packages:
 [1] Rgraphviz_2.30.0         graph_1.64.0             BiocGenerics_0.32.0      jpeg_0.1-8.1
 [5] broom_0.5.5              ggpubr_0.2.5             magrittr_1.5             kableExtra_1.1.0
 [9] superheat_0.1.0          viridis_0.5.1            viridisLite_0.3.0        scales_1.1.0
[13] forcats_0.5.0            dplyr_0.8.5              purrr_0.3.3              readr_1.3.1
[17] tidyr_1.0.2              tibble_2.1.3             tidyverse_1.3.0          ggcorrplot_0.1.3
[21] gridExtra_2.3            readxl_1.3.1             stringr_1.4.0            pheatmap_1.0.12
[25] ggcyto_1.14.1            ncdfFlow_2.32.0          BH_1.72.0-3              RcppArmadillo_0.9.850.1.0
[29] ggplot2_3.3.0            data.table_1.12.8        openCyto_1.24.0          flowWorkspace_3.34.1
[33] flowCore_1.52.1

loaded via a namespace (and not attached):
  [1] colorspace_1.4-1    ggsignif_0.6.0      ellipsis_0.3.0      rprojroot_1.3-2     mclust_5.4.5
  [6] corpcor_1.6.9       fs_1.3.2            clue_0.3-57         rstudioapi_0.11     farver_2.0.3
 [11] hexbin_1.28.1       remotes_2.1.1       IDPmisc_1.1.20      fansi_0.4.1         mvtnorm_1.1-0
 [16] lubridate_1.7.4     xml2_1.2.5          splines_3.6.2       R.methodsS3_1.8.0   mnormt_1.5-6
 [21] robustbase_0.93-5   knitr_1.28          pkgload_1.0.2       jsonlite_1.6.1      cluster_2.1.0
 [26] dbplyr_1.4.2        png_0.1-7           R.oo_1.23.0         BiocManager_1.30.10 rrcov_1.5-2
 [31] compiler_3.6.2      httr_1.4.1          backports_1.1.5     assertthat_0.2.1    Matrix_1.2-18
 [36] lazyeval_0.2.2      cli_2.0.2           prettyunits_1.1.1   htmltools_0.4.0     tools_3.6.2
 [41] gtable_0.3.0        glue_1.3.2          Rcpp_1.0.3          Biobase_2.46.0      cellranger_1.1.0
 [46] vctrs_0.2.4         nlme_3.1-145        xfun_0.12           ps_1.3.2            testthat_2.3.2
 [51] rvest_0.3.5         lifecycle_0.2.0     devtools_2.2.2      gtools_3.8.1        DEoptimR_1.0-8
 [56] zlibbioc_1.32.0     MASS_7.3-51.5       hms_0.5.3           RBGL_1.62.1         RColorBrewer_1.1-2
 [61] yaml_2.2.1          memoise_1.1.0       latticeExtra_0.6-29 stringi_1.4.6       desc_1.2.0
 [66] pcaPP_1.9-73        flowClust_3.24.0    pkgbuild_1.0.6      flowViz_1.50.0      rlang_0.4.5
 [71] pkgconfig_2.0.3     matrixStats_0.56.0  evaluate_0.14       fda_2.4.8.1         lattice_0.20-40
 [76] labeling_0.3        ks_1.11.7           processx_3.4.2      tidyselect_1.0.0    plyr_1.8.6
 [81] R6_2.4.1            generics_0.0.2      DBI_1.1.0           pillar_1.4.3        haven_2.2.0
 [86] withr_2.1.2         modelr_0.1.6        crayon_1.3.4        KernSmooth_2.23-16  ellipse_0.4.1
 [91] rmarkdown_2.1       usethis_1.5.1       callr_3.4.2         reprex_0.3.0        digest_0.6.25
 [96] webshot_0.5.2       R.utils_2.9.2       flowStats_3.44.0    RcppParallel_5.0.0  stats4_3.6.2
[101] munsell_0.5.0       sessioninfo_1.1.1
```

Supplementary Figure S6: Cyto-feature engineering pipeline session info. The R and package versions used for this manuscript are listed.

Cell Lineage	Associated Markers
Double Negative	CD3+ CD4- CD8-
T helper	CD3+ CD4+ CD8-
Cytotoxic T	CD3+ CD4- CD8+
Double Positive	CD3+ CD4+ CD8+

Supplementary Table S1: Definition of T cell lineages. The markers used to classify the T cell lineages are denoted in the "Associated Markers" column.

Cell Type	Associated Markers
Naive	CD44- CD62L+ Sca1-
Effector	CD44+ CD62L-
Central Memory	CD44+ CD62L+
Stem-cell Like Memory	CD44- CD62L+ Sca1+
Resident	CD103+

Supplementary Table S2: Definition of T cell types. The markers used to classify the T cell types are denoted in the "Associated Markers" column.

population	r squared	p-value	Adjusted p-value	Significance
Pop1	0.4979925	0.0001170	0.0003176	TRUE
Pop2	0.2974035	0.0058494	0.0085491	TRUE
Pop3	0.6881335	0.0000005	0.0000103	TRUE
Pop4	0.2587603	0.0111376	0.0156751	TRUE
Pop5	0.5633154	0.0000239	0.0001124	TRUE
Pop6	0.4265429	0.0005402	0.0012829	TRUE
Pop7	0.5294946	0.0000559	0.0001930	TRUE
Pop8	0.5988273	0.0000092	0.0000657	TRUE
Pop9	0.3932679	0.0010393	0.0019748	TRUE
Pop10	0.4185797	0.0006337	0.0014124	TRUE
Pop11	0.2480886	0.0132496	0.0179816	TRUE
Pop12	0.3704975	0.0015978	0.0028912	TRUE
Pop13	0.4738976	0.0002002	0.0005071	TRUE
Pop14	0.5063059	0.0000967	0.0002826	TRUE
Pop15	0.3377243	0.0029005	0.0045925	TRUE
Pop16	0.5346706	0.0000493	0.0001872	TRUE
Pop17	0.5098544	0.0000890	0.0002820	TRUE
Pop18	0.0979274	0.1365125	0.1621086	FALSE
Pop19	0.0025116	0.8161077	0.8381647	FALSE
Pop20	0.5592121	0.0000266	0.0001124	TRUE
Pop21	0.4033427	0.0008554	0.0017109	TRUE
Pop22	0.3465869	0.0024747	0.0040886	TRUE
Pop23	0.3172453	0.0041589	0.0063216	TRUE
Pop24	0.0487987	0.2995875	0.3162312	FALSE
Pop25	0.0602502	0.2476404	0.2688667	FALSE
Pop26	0.6502334	0.0000020	0.0000186	TRUE
Pop27	0.0003482	0.9310329	0.9310329	FALSE
Pop28	0.4158473	0.0006690	0.0014124	TRUE
Pop29	0.1353798	0.0769004	0.0942650	FALSE
Pop30	0.0816482	0.1758853	0.1965777	FALSE
Pop31	0.1622194	0.0510141	0.0668461	FALSE
Pop32	0.7600534	0.0000000	0.0000011	TRUE
Pop33	0.6721796	0.0000009	0.0000120	TRUE
Pop34	0.5765940	0.0000169	0.0000916	TRUE
Pop35	0.5944313	0.0000104	0.0000657	TRUE
Pop36	0.3640287	0.0018010	0.0031108	TRUE
Pop37	0.0955722	0.1415766	0.1630276	FALSE
Pop38	0.1514692	0.0601433	0.0761815	FALSE

Supplementary Table S3: Adjusted p-values for populations associated with bacterial burden. The r squared and p-values for the linear regression lines in Figure 5B are displayed for each population. The Benjamini and Hochberg False Discovery Rate correction is used to adjust the p-values based on the multiple tests that are performed. The significance column depicts if the adjusted p-value is less than 0.05.

T cell Panel – Surface

Fluor	Marker	Dilution	Catalog	Clone	RRID
BB515	Sca-1	1:1000	BD: 8127577	D7	RRID: AB_2739218
Alexa 532	CD3	1:50	Invitrogen: 58-0032-82	17A2	RRID: AB_11217479
PE Dazzle 594	CD62L	1:500	BioLegend: 104448	MEL-14	RRID: AB_2566163
PE Cy5	CD122	1:100	BioLegend: 123220	TM-β1	RRID: AB_2715962
PerCP Cy5.5	CD28	1:50	BioLegend: 102114	37.51	RRID: AB_2073850
PerCP e710	PD-1	1:100	Invitrogen: 46-9981-82	RMP1-30	RRID: AB_11151142
APC R700	CD103	1:200	BD: 565529	M290	RRID: AB_2739282
APC Fire750	CD44	1:1000	BioLegend: 103062	IM7	RRID: AB_2616727
BV480	CD4	1:100	BD: 565634	RM4-5	RRID: AB_2739312
BV570	CD8	1:100	BioLegend: 100739	53-6.7	RRID: AB_10897645
BV605	CTLA-4	1:50	BioLegend: 106323	UC10-4B9	RRID: AB_2566467
BV650	CD27	1:100	BioLegend: 124233	LG.3A10	RRID: AB_2687192
BV711	CD153	1:50	BD: 740751	RM153	RRID: AB_2740419
BV785	KLRG-1	1:100	BD: 565477	2F1	RRID: AB_2739256

T cell Panel – Intracellular

Fluor	Marker	Dilution	Catalog	Clone	RRID
PE	IL-17	1:100	BioLegend: 506904	TC11-18H10.1	RRID: AB_315464
PE Cy7	IFN-γ	1:100	Invitrogen: 25-7311-82	XMG1.2	RRID: AB_469680
BV421	IL-10	1:100	BioLegend: 505021	JESS-16E3	RRID: AB_10900417
Pacific Blue/e450	TNF-α	1:100	BioLegend: 506318	MP6-XT22	RRID: AB_893639

Supplementary Table S4: Flow cytometry antibodies. The T cell Surface panel antibody cocktail is prepared in FACS staining buffer with a 1:10 dilution of Brilliant Violet Buffer (BD). The T cell Intracellular panel antibody cocktail is prepared in permeabilization buffer.
